# Statin versus no statin after treatment with pipeline embolization device for intracranial aneurysms: a meta-analysis

**DOI:** 10.1055/s-0045-1809545

**Published:** 2025-06-21

**Authors:** Nathalia Soares Barbosa, Felipe Araujo Gouhie, Bezalel Hakkeem, Amanda Machado, João Paulo Mota Telles, Luís Gustavo Biondi Soares, Leandro de Assis Barbosa

**Affiliations:** 1 Escola Superior de Ciências da Santa Casa de Misericórdia de Vitória, Vitória ES, Brazil.; 2 Universidade Federal de Uberlândia, Uberlândia MG, Brazil.; 3 Jubilee Mission Medical College and Research Institute, Thrissur Kerala, India.; 4 Universidade Estadual Paulista, Botucatu SP, Brazil.; 5 Universidade de São Paulo, Departamento de Neurologia, São Paulo SP, Brazil.; 6 Hospital Estadual Central, Departamento de Neurocirurgia, Vitória ES, Brazil.

**Keywords:** Embolization, Therapeutic, Stents, Intracranial Aneurysm, Hydroxymethylglutaryl-CoA Reductase Inhibitors

## Abstract

**Background:**

Some studies demonstrated the role of statin therapy in improving outcomes after coil embolization or surgical clipping of cerebral aneurysm. However, the benefit of statins after pipeline embolization device (PED) for intracranial aneurysms is not well established.

**Objective:**

To evaluate the effects of statins on hemorrhagic and ischemic complications as well as on complete occlusion of aneurysm in the treatment with PED.

**Methods:**

We searched the PubMed, Embase, and Cochrane Library databases for articles published from their inception to November 2024. Data were collected from observational studies comparing statin to no statin therapy following pipeline embolization.

**Results:**

Four studies were included, comprising 2,822 patients and 3,063 aneurysms, 127 of which were ruptured and 4 of which received adjunctive coil embolization. Total hemorrhagic complication was reduced in the statin group (risk ratio [RR] = 0.50; 95%CI: 0.29–0.85; *p* = 0.010; I ^2^ = 0%) but did not reveal difference in restricted propensity score-matched (PSM) analysis (RR= 0.50; 95%CI: 0.24–1.07 *p* = 0.073; I ^2^ = 27%). There was no difference between the groups in complete occlusion of aneurysm rate at the last follow-up (RR = 0.94; 95%CI: 0.88–1.00; *p* = 0.055; I ^2^ = 8.0%) or total ischemic complications (RR = 1.48; 95%CI: 1.06–2.07; *p* = 0.021; I ^2^ = 0%).

**Conclusion:**

Statin use significantly reduced hemorrhagic complications after PED; however, this result should be interpreted cautiously due to study limitations. No significant differences were noted in complete occlusion rates or ischemic complications between the groups.

## INTRODUCTION

The pipeline embolization device (PED) is a widely used flow diverter in endovascular treatment 1 and also used in adjunctive coil. It covers a variety of intracranial aneurysms, such as large, giant, wide-necked, and fusiform since the main mechanism of action is the shunt of blood flow leading to intra-aneurysmal thrombosis in the long term. 2 To estimate the success of PED, it is necessary to calculate the rate of aneurysm occlusion; higher rates indicate a complete occlusion of aneurysm, especially in the last angiographic follow-up. 3

Currently, it has been demonstrated that statin therapy improves the outcome of coil embolization or surgical clipping of cerebral aneurysms, 4 and it is associated with aneurysm recurrence rate reduction, 5 possibly related to a benefit of statins in reducing inflammation and improving endothelial function outcomes in endovascular treatment. 6 7 However, there is no robust evidence about the impact of statin use after treatment with flow diverters.

For this reason, we aimed to conduct an updated systematic review and meta-analysis evaluating the efficacy of statin compared with not using it after pipeline embolization treatment for patients with intracranial aneurysms, exploring the complete occlusion of the aneurysm, as well as ischemic and hemorrhagic complications in the long term.

## METHODS

### Search strategy and data extraction

We systematically searched the PubMed, Embase, and Cochrane Library databases from their inception to November 2024 with the following search terms: *statin* , *Hypolipidemic Agents* , *Flow-diverter* , *stent* , *device* , *Pipeline embolization* , *PED* , *Brain aneurysms* , *Intracranial Aneurysm* , and *intracerebral aneurysm* . There were no language restrictions, and no other filters were used. The complete search strategy is detailed in **Supplementary Material Table S1** (available at https://www.arquivosdeneuropsiquiatria.org/wp-content/uploads/2025/04/ANP-2024.0384-Supplementary-Material.docx ). The references from all included studies, previous systematic reviews, and meta-analyses were also searched manually for any possible additional studies. The prospective meta-analysis protocol was registered on PROSPERO in December under protocol number CRD42024605398. Two authors (NB and AM) independently extracted the data using predefined criteria for search, data extraction, and quality assessment. Disagreements were resolved by consensus after discussing reasons for the discrepancy with a third author (BH).

### Eligibility criteria

Inclusion in the present meta-analysis was restricted to studies that met all the following eligibility criteria: randomized trials or observational studies; studies comparing statin use to no statin use; studies enrolling patients who underwent PED therapy for intracranial aneurysms; and studies reporting at least one outcome among the following: complete occlusion of aneurysm; stenosis of parent arteries; in-stent stenosis; ischemic complications, hemorrhage complications, all-cause mortality, neurologic mortality, and favorable (modified Rankin Scale [mRS] ≤ 1) and excellent (mRS ≤ 2) functional outcomes.

The exclusion criteria were case reports and studies involving endovascular treatment not using flow-diverter stent alone.

### Quality assessment

Two independent authors (AM and BH) evaluated the risk of bias assessment. Discrepancies were resolved through discussion with a third author (NB). All the studies in this meta-analysis were nonrandomized studies. It was evaluated with the Risk of Bias in Non-Randomized Studies-of Interventions (ROBINS-I). 8 In this scale, studies are scored as low, moderate, serious, or critical risk of bias in the domains of confounding, selection, interventions, missing data, measurement of outcomes and reporting of results. We also accessed the Grading of Recommendations Assessments, Development, and Evaluation 9 (GRADE) approach method and provided the “Summary of Findings” in **Supplementary Material Table S2** (online only).

### Endpoints and subanalysis

The selected endpoints were complete occlusion of aneurysm at the last follow-up and two composite outcomes: total ischemic complications and total hemorrhagic complications. Total ischemic complications included total ischemic complications, major ipsilateral ischemic stroke, ischemic stroke, and thromboembolic complications (symptomatic and asymptomatic). Total hemorrhagic complications covered total subarachnoid hemorrhage, major ipsilateral intracranial hemorrhage and hemorrhagic complications (symptomatic and asymptomatic). The amount of data outcomes available in the study was obtained from patients' aneurysms numbers. We conducted a subgroup analysis comparing Propensity Score-Matched (PSM) and non-PSM data in terms of complete aneurysm occlusion, overall ischemic complications, and overall hemorrhagic complications.

### Statistical analysis

The present systematic review and meta-analysis followed the Cochrane Collaboration Handbook of Systematic Review of Interventions and the Preferred Reporting Items for the Systematic Reviews and Meta-analysis (PRISMA) statement guidelines. 9 10

We employed risk ratio (RR) with 95%CIs as the measure of effect size to report binary outcomes. There were no continuous outcomes. Heterogeneity was assessed with the Cochran Q test and I ^2^ statistics. Values of *p <* 0.10 and of I ^2^ ≥ 25% were considered significant for heterogeneity. We used the Mantel-Haenszel random-effects model. All statistical analyses were performed using the R software (R Foundation for Statistical Computing), version 4.2.2.

### Sensitivity analyses

We conducted a subgroup analysis of studies reporting PSM data. In addition, we performed leave-one-out sensitivity analyses of the data with PSM to ensure the results were not dependent on a single study.

## RESULTS

### Study selection and characteristics

**Figure 1:**
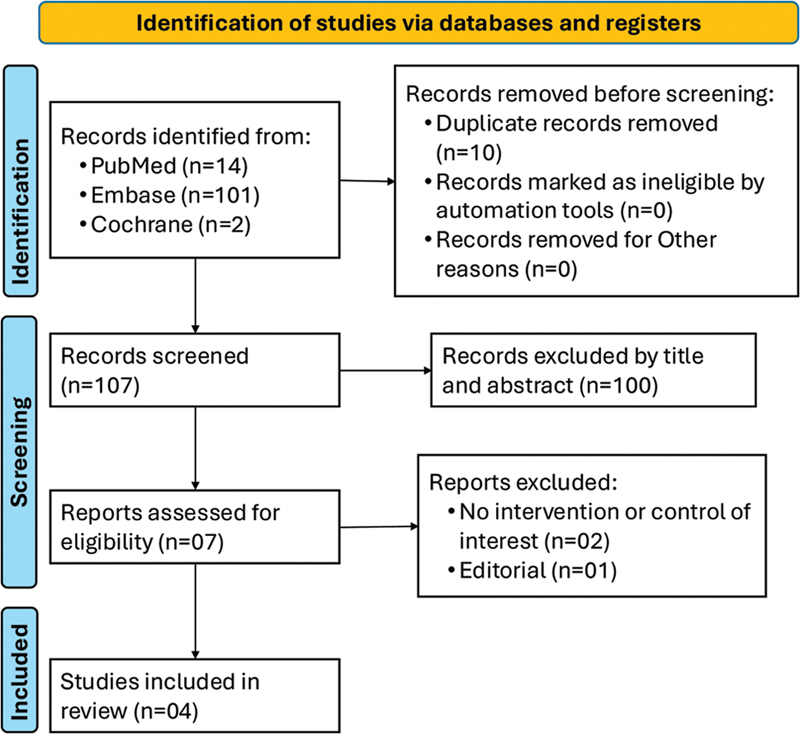
Preferred Reporting Items for Systematic Reviews and Meta-Analyses (PRISMA) flow diagram of study screening and selection.

The study selection process is illustrated in Figure 1 using the PRISMA flow diagram. Our systematic search yielded 117 potential articles. After removing duplicate reports and ineligible articles by title and abstract, seven studies were fully reviewed for inclusion and exclusion criteria. Finally, four observational studies 12 13 14 15 were included comprising a total of 2,822 patients, 643 of whom were on statin therapy and 2,179 who were not. The total number of aneurysms was 3,063, of which 127 were ruptured and only 4 received adjunctive coil embolization.

### Pooled analysis of included studies

**Figure 2:**
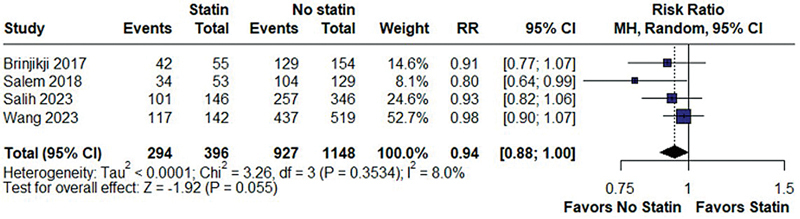
There was no significant difference between statin and no statin group in complete occlusion of aneurysm analysis at last follow-up.

**Figure 3:**
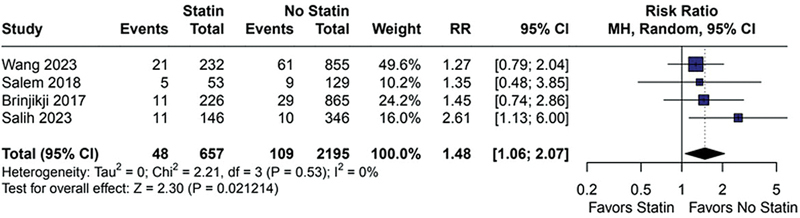
Total ischemic complications were not significantly different between statin and no statin.

**Figure 4:**
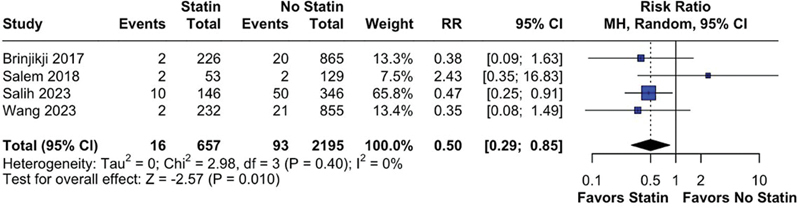
Statin reduced the risk of total hemorrhage complications after pipeline embolization device.

There was no statistically significant difference in the rates of complete occlusion of aneurysm at the last follow-up between the statin and non-statin groups (RR = 0.94; 95%CI: 0.88–1.00; *p* = 0.055; I ^2^ = 8.0%; Figure 2 ). Furthermore, there was no difference in the analysis of total ischemic complications (RR = 1.48; 95%CI: 1.06–2.07; *p* = 0.021; I ^2^ = 0%; Figure 3 ). Only total hemorrhagic complications analysis demonstrated significant statistical difference between the groups (RR = 0.50; 95%CI: 0.29–0.85; *p* = 0.010; I ^2^ = 0%; Figure 4 ).

#### Subanalysis of the selected population

A subanalysis was conducted considering the PSM results from Wang et al. 14 and Salih et al. 13 studies. We aimed to assess if the control of confounding factors could change the results of the meta-analysis. Nonetheless, the complete occlusion of aneurysm for PSM studies subgroup showed no difference (RR = 1.03; 95%CI: 0.85–1.26; *p* = 0.736; I ^2^ = 58%; **Figure S1** [online only]). The analysis not adjusted for propensity scores, in turn, found a statistical difference between the 2 groups (RR = 0.87; 95%CI: 0.76–0.99; *p* = 0.034; I ^2^ = 0%). There was no difference between subgroups *p* = 0.15.

The subgroup analysis for the total ischemic complications revealed no significant difference in both PSM (RR = 1.03; 95%CI: 0.32–3.34; *p* = 0.965; I ^2^ = 32%; **Figure S2** [online only]) and non-PSM groups (RR = 1.42; 95%CI: 0.80–2.51; *p* = 0.225; I ^2^ = 0%). The test analysis for the subgroups revealed no divergence among ischemic groups ( *p* = 0.63).

In contrast with the overall analysis of total hemorrhagic complications ( Figure 4 ), the subanalysis for hemorrhagic endpoints did not reveal any significant difference between statin and no statin in both PSM (RR= 0.37; 95%CI: 0.13–1.06; *p* = 0.063; I ^2^ = 7%; **Figure S3** [online only]) and non-PSM groups (RR= 0.86; 95%CI: 0.14–5.19; *p* = 0.868; I ^2^ = 56%). The complete analysis of both groups also did not reveal any difference (RR= 0.50; 95%CI: 0.24–1.07; *p* = 0.073; I ^2^ = 27%). There was no difference between subgroups *p* = 0.42.

#### Leave-one-out analysis

The total hemorrhagic complications leave-one-out analysis demonstrated that statin could prevent this event after omitting the Salem et al. 15 trial (RR = 0.38; 95%CI: 0.17–0.86; I ^2^ = 0%; **Figure S6** [online only]).

### Risk of bias assessment

The risk assessment of each study is presented in **Figure S7** (online only). All four studies were judged as moderate risk of bias, due to potential confounders inherent to observational studies, such as participant selection, classification of interventions, and deviation from intended interventions. Two studies were considered to have a moderate risk of bias due to missing data. 12 15 The GRADE approach for our study identified low certainty for complete aneurysm occlusion result as well as for total ischemic complications findings, and moderate certainty for total hemorrhage complications outcome, **Supplementary Material Table S2** (online only).

## DISCUSSION

In this systematic review and meta-analysis of 4 studies comprising 2,822 patients, we analyzed statins versus no statins for patients treated for intracranial aneurysms using PED. The findings in our review indicated that there was no significant effect of statin therapy for intracranial aneurysm in PED treatment regarding complete occlusion of aneurysm rate and total ischemic complications. However, our results indicated that statins may reduce the overall rate of hemorrhagic complications. In complete occlusion of aneurysm outcome, only in the analysis restricted to studies without PSM demonstrated statistical significance, which was not confirmed in the PSM-only and all-study analyses.

Statins have several cholesterol-independent effects called pleiotropic effects, which includes important mechanisms of vascular protection as antiinflammation, inhibition of vascular smooth muscle cell proliferation, anti-coagulation, and anti-oxidation. 15 Experimental studies indicate the same pathophysiology in cerebral aneurysms by improvement of endothelial function via inhibition of NF-κB activation which may have inhibitory effect on intracranial aneurysm progression, demonstrated on rats models. 16 17 However, it is well established that statins can reduce the absolute risk of ischemic strokes and others cardiovascular events 18 and, beyond that, provide better outcomes for patients undergoing carotid artery stenting in the long term and in the perioperative period. 19 20 21 Those facts point to the relevance of our study, which is the first meta-analysis to evaluate the impact of statin in cerebral aneurysm after flow diverter intervention.

The complete occlusion of aneurysm is the main goal of endovascular treatment. Many meta-analyses were performed to evaluate the efficacy of flow diverters and demonstrated a high rate of aneurysm occlusion, varying according to aneurysm location, as posterior circulation and size greater than 7 mm were associated with low oclusion rate; 22 also, in a large versus small analysis, the long-term occlusion rate after PED treatment was higher in the small-size group. 23 On the other hand, a meta-analysis by Gaith et al., who studied a large sample of aneurysm treated with PED, concluded that aneurysm occlusion rates were high regardless of size. 24

Although some trials indicated factors such as increasing aneurysm neck diameter and treatment before PED deployment 25 as predictors of incomplete occlusion, as well as the follow-up time, due the aneurysm's resolution occurs gradually, wherefore a shorter or longer follow-up may influence in complete occlusion rate, 26 the baseline characteristics ( Table 1 ) from our studies do not reveal significant differences between the intervention and control group in relation to those factors previous presented. Furthermore, device mispositioning, inadequate coverage of the aneurysm neck, and inclusion of a branch vessel into the aneurysm fundus are also associated with the success of the flow diverter, 27 which cannot be controlled by statin use. This maybe reinforces the insignificant result for occlusion outcome in our meta-analysis. Even though the non-PSM subanalysis indicated a statically favorable difference for treatment with statin, caution is needed to interpret this result, indicating the need for new clinical trials to adequately support it.

Ischemic endpoints rarely occur in intracranial aneurysms in natural history. Their origin can be from embolization of a luminal thrombus ejected by an aneurysm to distal vessels, extension of a thrombus in the aneurysmal sac into the parent artery's lumen, and aneurysmal mass effect. 28 As previously discussed, statins' potential antiinflammatory actions stabilize atheromatous plaques in the carotid artery and coronary artery atherosclerosis. But this mechanism does not seem to be associated with thrombogenic pathways from intracranial aneurysms treated by PED, owing to come from thrombogenic materials in flow diverters constitution, misposition or device's movement, and resistance to antiplatelet agents. 29

Ischemic events are valso frequently related to anterior circulation, which presented the highest prevalence in our study, in both the statin and non-statin groups; besides, small and large unruptured aneurysms are vulnerable to this endpoint. 30 These findings and the lack of statistical relevance from our analysis suggest that the efforts to prevent outcomes such as ischemic stroke and thromboembolic complications may be directed to other strategies. Even if statin patients in the hyperlipidemic group are more likely to have ischemic injuries, not all patients from the studies included were statin users before the procedure. Wang et al. 14 could observe the effect of statin initiation postprocedure and include the Asian population, who have a higher risk of atherosclerosis compared with the Caucasian population. Nevertheless, they did not observe any statistical difference in total ischemic complications, corroborating with our result.

Other risk factors related to patient's characteristics were pointed out as predictable factors for ischemic stroke after pipeline embolization. Brinjikji et al. concluded that fusiform aneurysms were the only variable independently associated with postoperative stroke. In contrast, our review, in general, covered a low number of fusiform aneurysms compared with saccular aneurysms, which may not contribute to statistical strength. 31

Hemorrhagic complications such as subarachnoid and intraparenchymal hemorrhage are rare and devasting endpoints post-PED procedures. The results of the meta-analysis by Brinjikji et al. 24 suggest that PED has a higher risk of subarachnoid hemorrhage in large and giant aneurysms, which were more prevalent in our study. According to our results, this may indicate that statins could be a potential protector for bleeding events in stent diverter postprocedure. However, the PSM subanalysis did not reveal statistical differences and indicates a probable effect of confounding factors on the results found in raw data. The leave-one-out analysis omitting the results of the study by Salem et al. 15 also supports the effect of statins in the prevention of hemorrhagic complications. However, the omission of this piece of data should be interpreted with caution since the effect of statins appears stronger when a single study is removed, which may indicate that more robust studies are necessary to establish this outcome.

Finally, flow-diverting stents are currently experiencing potential growth, but, even though they have been proven to be a safe and effective technique, they are not free from serious complications. In addition, there is a lack of comparative studies evaluating coil embolization and flow-diverter stents in the context of statin therapy. This represents a clinically relevant gap, considering that statin use has been associated with decreased aneurysm recurrence following coil embolization. 5 32 33 34 For this reason, larger and randomized studies are necessary to evaluate the potential of statins to improve post-PED outcomes, including hemorrhagic complications, which statins demonstrated to prevent.

### Limitations

Our study has several limitations that need to be considered. First, there are only a few studies that have evaluated the use of statins in patients undergoing PED treatment, and all of them were observational studies. Although we needed to include observational studies that contributed to great insights, they are not the strongest source of evidence given that the findings were affected by possible publication bias. As an example, the PSM data changed the results of hemorrhagic outcome, balancing confounding factors and revealing a weakness of evidence in our results. Moreover, the studies vary in terms of duration of treatment, follow-up time and, although most studies do not mention, probably the intensity of statins was also different.

In conclusion, due to the limitations of a meta-analysis composed of a small number of observational studies, new randomized controlled trials (RCTs) need to be conducted to enhance confidence in our finding that statins maybe prevent hemorrhagic complications after PED in intracranial aneurysm treatment. There was no difference between statin and no-statin treatment in complete occlusion of aneurysm and total ischemic complications.

**Table 1:** Articles' information and population characteristics

	Brinjikji et al. 12 (2017)		Salem et al. 15 (2018)		Salih et al. 13 (2023)		Wang et al. 14 (2023)	
	Statin	Non statin	Statin	Non statin	Statin	No statin	Statin	Non statin
Study design	Post-hoc analysis ^a^		Retrospective observational cohort		Retrospective observational cohort PSM		Retrospective cohort observational PSM	
Nr. of patients (n)	226	866	39	112	49	49	188	188
Female (%)	84.1	80.6	75.4	87.5	81.6	81.6	68.6	72.3
Age ^†^	64.6 ± 9.6	55.5 ± 14.0	67* (62–70)	56* (46–62)	59.3 ± 9.8	59.3 ± 10	55.46 ± 10.43	54.77 ± 10.37
Smoking history (%)	NA	NA	30.2	19.4	14.3	20.4	27.6	25.5
Hypertension (%)	75.5	41.1	NA	NA	NA	NA	45.7	43.6
Diabetes (%)	NA	NA	NA	NA	8.2	4.1	8.0	8.0
Ruptured aneurysm (%)	4.5	6.7	0	5.4	NA	NA	3.7	4.3
Aneurysm size ^†^ (mm)	11.6 ± 7.0	12.1 ± 8.0	NA	NA	NA	NA	11.8 ± 8.6	14.0 ± 8.8
Small/medium aneurysm (%)	36.4	39.9	NA	NA	NA	NA	51.1	48.9
Large aneurysm (%)	54.8	50.0	NA	NA	NA	NA	49.0	50.9
Giant aneurysm (%)	8.8	10.1	NA	NA	NA	NA	7.4	7.9
Aneurysm neck ^†^ (mm)	6.6 ± 4.9	6.6 ± 4.8	NA	NA	4.1 ± 2.4	3.9 ± 2	5.98 ± 4.03	6.70 ± 3.99
Saccular aneurysm (%)	70.9	76.3	NA	NA	89.8	89.8	81.4	84.0
Fusiform aneurysm (%)	20.8	14.9	NA	NA	10.2	10.2	7.4	9.0
Anterior circulation (%)	84.5	83.6	81.1	89.2	87.7	81.6	86.7	88.8
Posterior circulation (%)	4.5	5.7	18.9	10.8	12.2	18.4	13.3	11.2
Follow-up (Months)	22.1 ± 15.1 (clinical) 28.3 ± 23.7 (angiographic)		7.2		23.8 ± 19.7	20 ± 14.5	3 years	

Abbreviations: NA, not available; PSM, propensity score-matched.

Notes: ^†^ Mean; ^*^ median (interquartile range, IQR); ^a^ post-hoc analysis from pooled patient-level datasets from 3 pipeline embolization device studies viz prospective single-arm clinical trial (PUFS) retrospective postmarket registry (IntrePED) and prospective postmarket registry (ASPIRe); Salem et al. provided all data calculated based on the number of aneurysms; Brinjikji et al. provided the angiographic data calculated based on the number of aneurysms and clinical data calculated based on the number of patients.
